# Sexual well-being in old age

**DOI:** 10.1192/j.eurpsy.2021.1124

**Published:** 2021-08-13

**Authors:** S. Von Humboldt, I. Leal

**Affiliations:** William James Center For Research, ISPA-Instituto Universitário, Lisbon, Portugal

**Keywords:** multiple correspondence analysis, sexual well-being, Portuguese older adults, English older adults

## Abstract

**Introduction:**

Older adults who engage in sexual activities may benefit from increasing psychological and physical well-being, which may contribute to reduce a number of physical and mental health problems.

**Objectives:**

To analyze sexual well-being (SWB) in older adults’ perspective and to examine the potential explanatory mechanisms of a SWB overall model, in an older cross-national sample.

**Methods:**

Measures were completed, using a variety of appropriate methods, including demographics and interviews. Complete data were available for 326 older adults aged between 65-102 years. Data were subjected to content analysis. Representation of the associations and latent constructs were analyzed by a Multiple Correspondence Analysis (MCA).

**Results:**

The most prevalent response of the interviewed participants for SWB was “touching and caring” (18.0%). A three-dimension model formed by “care and well-being”, “attractiveness, intimacy and touching”, and “sexual intercourse and pleasure” was presented as a best-fit solution for English older adults. SWB for Portuguese older adults were explained by a three-factor model: “health and desire”, “care, eroticism and affection” and “penetration sex”.

**Conclusions:**

The outcomes presented in this paper emphasized the need to explore the diversity of indicators of SWB among older adults and the cultural differences of a SWB model for older adults.

